# The Prognostic Significance of the Fibrinogen-to-Albumin Ratio in Patients With Triple-Negative Breast Cancer: A Retrospective Study

**DOI:** 10.3389/fsurg.2022.916298

**Published:** 2022-06-14

**Authors:** Qinheng Yang, Dong Liang, Yang Yu, Feng Lv

**Affiliations:** Department of Breast Surgery, Henan Provincial People’s Hospital; Zhengzhou University People’s Hospital; Henan University People’s Hospital, Zhengzhou, China

**Keywords:** fibrinogen-to-albumin ratio, triple-negative breast cancer, fibrinogen, albumin, chemotherapy

## Abstract

**Objective:**

This study aims to investigate the potential prognostic value of fibrinogen-to-albumin ratio (FAR) in patients with triple-negative breast cancer (TNBC).

**Methods:**

This study used a retrospective design and enrolled 224 patients with TNBC treated between January 2009 and December 2014 at the Henan Provincial People’s Hospital. The receiver operating characteristic curve (ROC) was used to determine the optimal cut-off value for FAR. The associations between TNBC and clinicopathologic categorical variables by FAR were analyzed using the Chi-square test or Fisher’s exact test. The survival time and survival curve were determined by Kaplan-Meier survival analysis and compared using the Log-rank method. The potential prognostic factors were determined using univariate and multivariate Cox proportional hazard regression models. Prognostic nomogram was established on the basis of the multivariate analyses. The calibration curves were used to assess the predictive performance.

**Results:**

The optimal cut-off value for FAR based on the overall survival (OS) was 0.066, as evaluated by the ROC. The 224 included patients were divided into low FAR group (<0.066) and high FAR group (≥0.066). Univariate and multivariate models shown that FAR was an potential prognostic factor for disease-free survival (DFS) and OS in patients with TNBC. The median DFS and OS of the low FAR group were longer than those of the high FAR group (*χ*^2 ^= 15.080, *P* = 0.0001; *χ*^2 ^= 13.140, *P* = 0.0003), including for pre-menopausal patients, and those with pathological stages I + II, and lymph vessel invasion. A nomogram based on the potential prognostic factors was efficient in predicting 3-, and 5-year DFS and OS survival probabilities.

**Conclusions:**

The FAR, which is tested routinely and is characterized by its simplicity, objectivity, and inexpensiveness, is a potential prognostic factor of TNBC, and is potentially applicable in clinical practice.

## Introduction

Breast cancer is the most common malignant tumor in females, particularly among the aging population, and its incidence rate is increasing yearly, and has become an important cause of cancer-related morbidity and mortality worldwide (1). In the 2020 global statistics, there were 19.29 million new cancer cases including 9.23 million female cases; and of the 2.26 million new breast cancer cases, about 680,000 died (2). Moreover, from the China National Cancer Center data, of the 272,400 new cancer cases, about 70,700 died (3, 4). Moreover, western lifestyle and dietary changes are the important reasons for the rapid rise in breast cancer disease burden (3, 4). Triple-negative breast cancer (TNBC) is defined by the absence of estrogen receptor (ER), progesterone receptor (PR) and human epidermal growth factor receptor-2 (HER2), and accounts for 20% of all invasive breast cancers (5). Furthermore, TNBC is characterized by high invasiveness, easy recurrence and metastasis, and poor prognosis; and it is not sensitive to traditional endocrine or targeted therapy (6).

It has been reported that tumor-associated inflammatory response (TAIR) has a critically important role in the occurrence, development of lymph node metastasis, treatment, and prognosis of tumors, and has received increasing attention from researchers (7, 8). In the occurrence and process of development of malignant tumors, coagulation dysfunction occurs with tumors, which increases the risk of thrombosis (9). Thrombosis promotes the proliferation, invasion, and metastasis of malignant tumor cells by inhibiting the functions of growth factors and natural killer cells (NK cells) (10, 11). Fibrinogen (FIB) and albumin (ALB) have attracted wide attention as noninvasive prognostic factors of various cancers. FIB is a soluble serum glycoprotein synthesized by hepatocytes, and participates in blood coagulation and platelet aggregation (12). Hypercoagulable state, acute infection, and malignant tumor can cause changes in plasma FIB levels, and the plasma FIB is driven by interleukin and cytokines (13). ALB is an important factor of nutritional status, and hypoproteinemia is a reliable indicator of malignant tumor cachexia and malnutrition (14). Moreover, malnutrition is often accompanied by autoimmune dysfunction, which can accelerate the replication and proliferation of tumor cells, and also reflects systemic inflammatory response in patients with tumor (15). Furthermore, the FAR, based on FIB and ALB, has gained credibility as a promising inflammation-based prognostic indicator in various solid tumors (16–18). Although low FAR is found to be associated with poor survival in breast cancer; however, FAR has rarely been studied in TNBC patients. Therefore, our study aimed to investigate the prognostic significance of FAR in patients with TNBC and provide a reference for the treatment of TNBC.

## Materials and Methods

### Ethics Approval and Informed Consent

The study design was approved by the Ethics Committee of Henan Provincial People’ s Hospital. Written informed consent was obtained from all enrolled participants. This study complied with the standards of the Declaration of Helsinki and its subsequent amendments or similar ethical standards.

### Study Population

**Figure 1:**
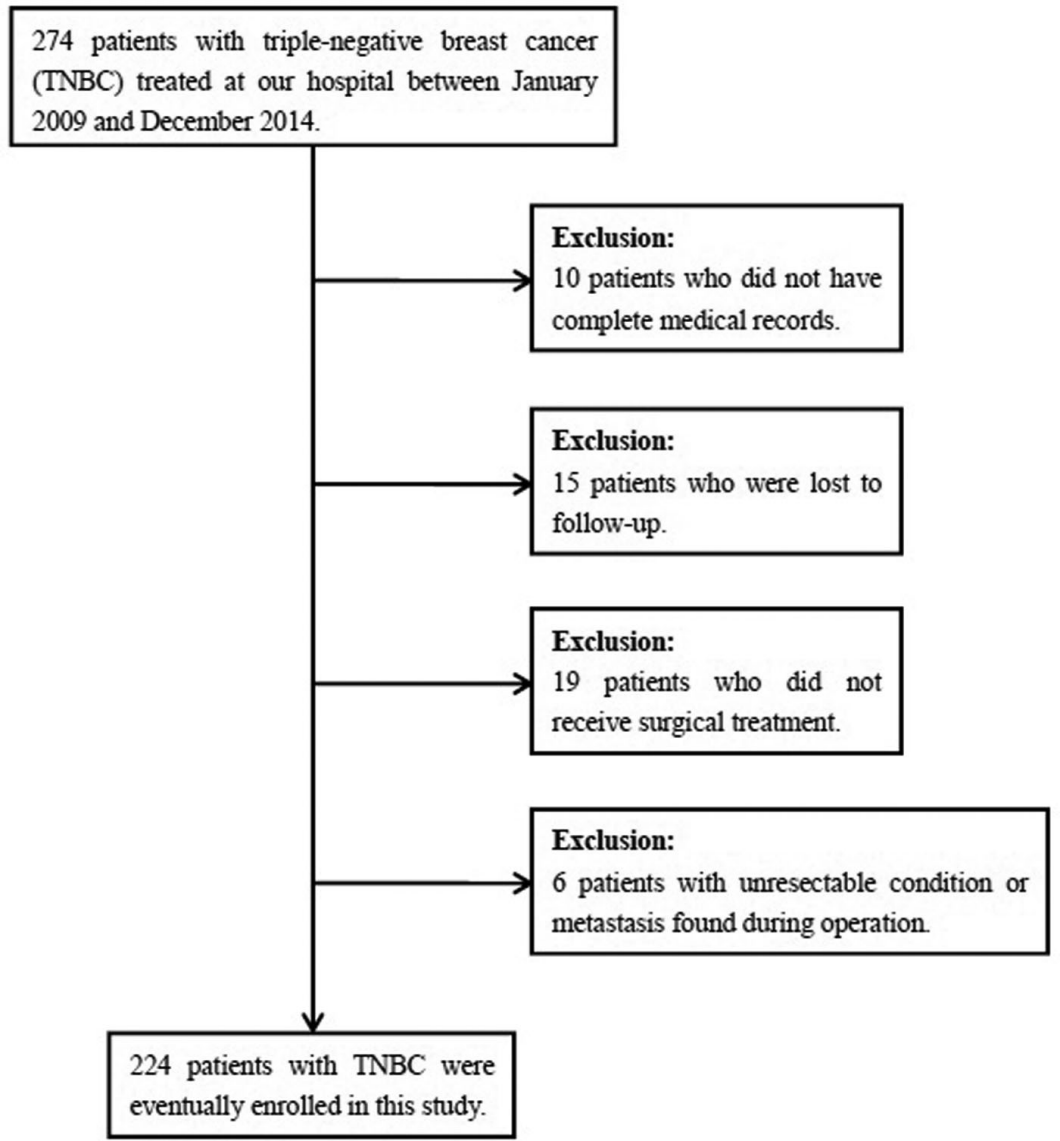
Flow diagram of the process of selection of the patients included in this study.

In total, 274 patients with TNBC were treated at Henan Provincial People’ s Hospital between January 2009 and December 2014. According to the inclusion and exclusion criteria, 224 patients were eventually included, while the remaining 70 patients were excluded (Figure 1). All patients were histologically-confirmed. Clinicopathologic features, detailed treatment, and follow-up data of the patients were extracted from the medical records.

### Inclusion Criteria and Exclusion Criteria

The following inclusion criteria were used to select patients who: (1) were confirmed by pathology and classified as TNBC subtype; (2) had complete medical records and follow-up information; (3) Karnofsky performance status (KPS) scores ≥80 and Zubrod-Eastern Cooperative Oncology Group (ECOG)-WHO <2; (4) pathological TNM stages I–III; and (5) the blood samples were collected prior to the treatment. The following patients met the exclusion criteria: (1) those with synchronous, metachronous tumors, or distant metastases; (2) those with acute or chronic inflammatory diseases (hypertension, diabetes, and immune system diseases); (3) those receiving antitumor therapy; and (4) those who were administered anti-inflammatory medications.

### Evaluation

The TNM stage classification was according to the eighth edition of the American Joint Committee on Cancer (AJCC) and Union for International Cancer Control (UICC) (19, 20). Hematoxylin and eosin (HE) staining was performed to detect lymph vessel invasion (LVI) and neural invasion in breast cancer tissues.

### Peripheral Venous Blood Parameters

Peripheral venous blood was collected at definite time points before treatment. FAR’s definition was as follows: FAR = [FIB/ALB], where FIB and ALB were pretreatment peripheral FIB and ALB counts, respectively.

### Follow up

All patients were followed-up regularly by telephone, or as inpatients or outpatients. The postoperative schedule included reexamination every three months for the first and second years, every six months for the third through fifth year, and then at twelve-monthly intervals thereafter. The following follow-up procedures were performed: clinical examination with laboratory tests (routine blood tests, blood biochemistry), ultrasonography of the breast, mammography, and other examinations. Disease-free survival (DFS) was defined as the time from surgery to recurrence or progression, death from any cause, or the last follow-up while overall survival (OS) was the time from surgery to death or last follow-up.

### Statistical Analysis

All statistical analyses were performed using IBM SPSS Statistics for Windows, version 22.0 (IBM Corp., Armonk, NY, USA), GraphPad Prism software, version 8.0 (La Jolla, CA, USA) and R (version 3.6.0; Vienna, Austria. URL: http://www.R-project.org/). The receiver operating characteristic (ROC) curve was used to determine the optimal cutoff value of FAR, based on which FAR was categorized into two groups. Clinicopathologic categorical variables were analyzed using the Chi-square test or Fisher’s exact test. The Kaplan-Meier method and Log-rank test were used to determine the survival time and survival curve. Univariate and multivariate Cox proportional hazards regression models were used to evaluate the independent prognostic factors. The hazard ratio (HR) and 95% confidence intervals (CIs) for the risk of recurrence associated with FAR and breast cancer prognosis were calculated. Prognostic nomogram was established according to the multivariate analyses. The calibration curve was used to assess the predictive performance. Statistical significance was set at *P* < 0.05.

## Results

### Patient Clinicopathologic Characteristics

The demographic and clinicopathologic characteristics of the all female, 224 pathologically-diagnosed patients with TNBC were summarized in Table 1. The median age was 46 years, and ranged from 25 to 72 years. Of the 224 enrolled patients, 211 cases were married, 13 cases were unmarried; and 131 cases were premenopausal, 93 cases were postmenopausal, respectively. The patients were stratified into two groups: the low FAR (<0.066) group and high FAR (≥0.066) group, according to the optimal FAR cutoff value of 0.066, evaluated by ROC. There were 126 (56.25%) cases in low FAR group and 98 (43.75%) cases in high FAR group, respectively. Significant differences occurred with being menopausal (*χ*^2 ^= 13.241, *P* = 0.0003).

**Table 1:** Demographic and clinicopathologic characteristics in triple-negative breast cancer.

Parameters		Low FAR (*N* = 126)	High FAR (*N* = 98)	*χ* ^2^	*P-*value
Age (years)				0.010	0.919
<46	106	60	46		
≥46	118	66	52		
Marital status				0.157	0.692
Married	211	118	93		
Unmarried	13	8	5		
BMI				0.005	0.946
<24.00	108	61	47		
≥24.00	116	65	51		
Family history				1.422	0.233
Yes	64	40	24		
No	160	86	74		
Menopause				13.241	0.0003
Yes	93	39	54		
No	131	87	44		
Tumor site				0.180	0.671
Right	97	53	44		
Left	127	73	54		
Histologic type				0.014	0.907
Ductal	213	120	93		
Lobular	11	6	5		
Histologic grade				0.950	0.330
I + II	165	96	69		
III	59	30	29		
Postoperative complications				2.461	0.117
Yes	16	6	10		
No	208	120	88		
Post-chemotherapy				0.139	0.710
Yes	147	84	63		
No	77	42	35		
Post-radiotherapy				0.925	0.336
Yes	181	99	82		
No	43	27	16		
Adverse effects of radiotherapy				0.009	0.925
Yes	61	34	27		
No	163	92	71		

*FAR, fibrinogen-to-albumin ratio*.

### The Correlations Between FAR and Pathological Data in TNBC

Overall, 167 and 57 patients underwent total mastectomy and breast-conserving surgery, respectively. With respect to pathological TNM stage at diagnosis, 119 (53.13%) and 105 (46.87%) patients with breast cancer had stages I + II and III disease, respectively. There were significant differences in neural invasion between the two groups (*χ*^2 ^= 5.236, *P* = 0.022). Table 2 shown the detailed information.

**Table 2:** The correlations between FAR and pathological data in triple-negative breast cancer.

Parameters		Low FAR (*N* = 126)	High FAR (*N* = 98)	*χ* ^2^	*P-*value
Type of surgery				0.359	0.549
Breast-conserving surgery	57	34	23		
Mastectomy	167	92	75		
Pathological T stage				0.0003	0.987
T1 + T2	103	58	45		
T3 + T4	121	68	53		
Pathological N stage				0.209	0.647
N0 + N1	125	72	53		
N2 + N3	99	54	45		
Pathological TNM stage				1.202	0.273
I + II	119	71	48		
III	105	55	50		
P/T ratio				1.429	0.232
<0.12	113	68	45		
≥0.12	111	58	53		
Total lymph nodes				0.385	0.535
<20	101	60	41		
≥20	123	68	55		
Positive lymph nodes				0.727	0.394
<2	94	56	38		
≥2	130	70	60		
Ki-67 status				0.004	0.949
Negative (≤14%)	43	24	19		
Positive (>14%)	181	102	79		
CK status				0.056	0.814
Negative	209	118	91		
Positive	15	8	7		
E-cad status				1.833	0.176
Negative	65	32	33		
Positive	159	94	65		
EGFR status				1.527	0.217
Negative	194	106	88		
Positive	30	20	10		
P53 status				1.185	0.276
Negative	96	50	46		
Positive	128	76	52		
Lymph vessel invasion				0.046	0.831
Negative	148	84	64		
Positive	76	42	34		
Neural invasion				5.236	0.022
Negative	180	108	72		
Positive	44	18	26		

*FAR, fibrinogen-to-albumin ratio; EGFR, epidermal growth factor receptor.*

### Associations Between FAR and Inflammation Indexes

Regarding blood parameters collected before surgery, there were significant differences in ALB (*χ*^2 ^= 25.244, *P* < 0.0001), FIB (*χ*^2 ^= 143.300, *P* < 0.0001), alkaline phosphatase (ALP) (*χ*^2 ^= 11.936, *P* = 0.001), and fibrin degradation products (FDPs) (*χ*^2 ^= 4.644, *P* = 0.031). Associations between the FAR and inflammation indices were shown in Table 3.

**Table 3:** Associations between FAR and inflammation indices.

Parameters		Low FAR (*N* = 126)	High FAR (*N* = 98)	*χ* ^2^	*P-*value
ALB				25.244	<0.0001
<45.00	106	41	65		
≥45.00	118	85	33		
FIB				143.300	<0.0001
<2.90	113	108	5		
≥2.90	111	18	93		
ALT				0.014	0.906
<14.00	111	62	49		
≥14.00	113	64	49		
AST				2.568	0.109
<17.00	105	65	40		
≥17.00	119	61	58		
ALP				11.936	0.001
<63.00	107	73	34		
≥63.00	117	53	64		
≥1.10	107	60	47		
CEA				0.092	0.762
<1.59	114	63	51		
≥1.59	110	63	47		
CA125				1.161	0.281
<12.75	112	59	53		
≥12.75	112	67	45		
CA153				0.431	0.511
<11.50	113	66	47		
≥11.50	111	60	51		
FDP				4.644	0.031
<1.25	112	71	41		
≥1.25	112	55	57		

*FAR, fibrinogen-to-albumin ratio; ALB, albumin; FIB, fibrinogen; ALT, alanine transaminase; AST, aspartate transaminase; ALP, alkaline phosphatase; CEA, carcinoembryonic antigen; CA, cancer antigen; FDP, fibrin degradation product.*

### Univariate and Multivariate Cox Regression Survival Analyses

The optimal cutoff value of 0.066 for FAR was significantly correlated with DFS and OS. Univariate and multivariate Cox regression survival analyses revealed that family history, FAR, pathological T stage, total lymph nodes, E-cadherin (E-cad) status, LVI, neural invasion, and postoperative chemotherapy were significant prognostic factors for DFS; while family history, FAR, pathological T stage, total lymph nodes, E-cad status, LVI, neural invasion, and postoperative chemotherapy were significant prognostic factors for OS. The results were presented in Table 4. And the results were displayed using forest plots, and shown in Supplementary Figure S1.

**Table 4:** Univariate and multivariate Cox regression survival analyses of the FAR for the prediction of DFS and OS in triple-negative breast cancer.

Parameters	DFS				OS			
	Univariate analysis		Multivariate analysis		Univariate analysis		Multivariate analysis	
Cases (*n*)	Hazard ratio (95%CI)	*p* value	Hazard ratio (95%CI)	*p* value	Hazard ratio (95%CI)	*p* value	Hazard ratio (95%CI)	*p* value
Age (years)		0.098				0.631		
<46	1(Reference)				1(Reference)			
≥46	1.867(0.892–3.906)				1.180(0.600–2.321)			
Marital status		0.941				0.261		
Married	1(Reference)				1(Reference)			
Unmarried	1.055(0.254–4.378)				0.435(0.102–1.858)			
BMI		0.624				0.512		
<24.00	1(Reference)				1(Reference)			
≥24.00	1.222(0.549–2.716)				1.305(0.590–2.887)			
Family history		0.030		0.002		0.004		<0.0001
Yes	1(Reference)		1(Reference)		1(Reference)		1(Reference)	
No	0.412(0.185–0.919)		0.268(0.115–0.628)		0.294(0.126–0.683)		0.222(0.095–0.518)	
Menopause		0.597				0.872		
Yes	1(Reference)				1(Reference)			
No	1.249(0.547–2.855)				1.078(0.430–2.707)			
FAR		0.0001		<0.0001		0.0006		<0.0001
<0.066	1(Reference)		1(Reference)		1(Reference)		1(Reference)	
≥0.066	3.395(1.763–6.538)		4.600(2.201–9.616)		3.166(1.641–6.111)		5.555(2.472–12.481)	
ALB		0.665				0.213		
<45.00	1(Reference)				1(Reference)			
≥45.00	0.832(0.362–1.912)				0.574(0.239–1.376)			
ALT		0.660				0.075		
<14.00	1(Reference)				1(Reference)			
≥14.00	1.205(0.525–2.764)				2.229(0.922–5.389)			
AST		0.120				0.044		
<17.00	1(Reference)				1(Reference)			
≥17.00	1.842(0.853–3.975)				2.377(1.025–5.510)			
ALP		0.363				0.155		
<63.00	1(Reference)				1(Reference)			
≥63.00	1.366(0.697–2.675)				1.785(0.803–3.970)			
CEA		0.753				0.550		
<1.59	1(Reference)				1(Reference)			
≥1.59	1.104(0.597–2.042)				1.216(0.640–2.310)			
CA125		0.536				0.126		
<12.75	1(Reference)				1(Reference)			
v≥12.75	1.651(0.337–8.089)				1.681(0.864–3.272)			
CA153		0.360				0.092		
<11.50	1(Reference)				1(Reference)			
≥11.50	1.362(0.702–2.642)				1.865(0.903–3.849)			
FIB		0.085				0.792		
<2.90	1(Reference)				1(Reference)			
≥2.90	2.343(0.889–6.172)				1.143(0.425–3.073)			
FDP		0.111				0.225		
<1.25	1(Reference)				1(Reference)			
≥1.25	1.925(0.860–4.307)				1.754(0.708–4.348)			
Tumor site		0.611				0.055		
Right	1(Reference)				1(Reference)			
Left	1.591(0.266–9.526)				2.113(0.983–4.544)			
Type of surgery		0.830				0.192		
Breast-conserving surgery	1(Reference)				1(Reference)			
Mastectomy	1.127(0.378–3.364)				2.057(0.696–6.080)			
Tumor size		0.685				0.429		
≤2cm	1(Reference)				1(Reference)			
> 2cm	1.288(0.379–4.380)				1.712(0.451–6.491)			
Histologic type		0.488				0.340		
Ductal	1(Reference)				1(Reference)			
Lobular	1.838(0.329–10.262)				1.749(0.291–9.009)			
Histologic grade		0.570				0.499		
I + II	1(Reference)				1(Reference)			
III	1.245(0.585–2.651)				1.335(0.578–3.082)			
Pathological T stage		0.023		0.036		<0.0001		0.002
T1 + T2	1(Reference)		1(Reference)		1(Reference)		1(Reference)	
T3 + T4	2.354(1.128–4.913)		1.665(1.035–2.680)		1.486(1.037–5.791)		1.582(1.053–6.969)	
Pathological N stage		0.108				0.253		
N0 + N1	1(Reference)				1(Reference)			
N2 + N3	5.651(0.684–46.690)				3.817(0.385–37.886)			
Pathological TNM stage		0.096				0.100		
I + II	1(Reference)				1(Reference)			
III	5.983(0.730–49.012)				5.685(0.716–45.168)			
P/T ratio		0.601				0.172		
<0.12	1(Reference)				1(Reference)			
≥0.12	1.554(0.298–8.100)				3.245(0.598–17.596)			
Total lymph nodes		0.009		0.019		0.019		0.004
<20	1(Reference)		1(Reference)		1(Reference)		1(Reference)	
≥20	1.812(1.158–2.834)		1.790(1.100–2.912)		2.391(1.151–4.965)		2.042(1.262–3.303)	
Positive lymph nodes		0.470				0.525		
<2	1(Reference)				1(Reference)			
≥2	1.488(0.507–4.366)				1.485(0.438–5.036)			
Ki-67 status		0.538				0.466		
Negative (≤14%)	1(Reference)				1(Reference)			
Positive (>14%)	1.347(0.521–3.483)				1.449(0.535–3.927)			
CK status		0.934				0.248		
Negative	1(Reference)				1(Reference)			
Positive	1.068(0.228–5.002)				2.409(0.542–10.697)			
E-cad status		0.001		0.043		0.001		0.036
Negative	1(Reference)		1(Reference)		1(Reference)		1(Reference)	
Positive	0.021(0.002–0.202)		0.088(0.008–0.923)		0.248(0.105–0.585)		0.564(0.329–0.965)	
EGFR status		0.587				0.896		
Negative	1(Reference)				1(Reference)			
Positive	1.362(0.447–4.154)				1.077(0.352–3.293)			
P53 status		0.305				0.295		
Negative	1(Reference)				1(Reference)			
Positive	1.489(0.696–3.182)				1.511(0.698–3.271)			
Lymph vessel invasion		<0.0001		<0.0001		<0.0001		<0.0001
Negative	1(Reference)		1(Reference)		1(Reference)		1(Reference)	
Positive	4.992(2.295–10.858)		3.865(2.237–6.681)		5.582(2.374–13.125)		4.893(2.675–8.950)	
Neural invasion		0.010		0.002		0.002		0.002
Negative	1(Reference)		1(Reference)		1(Reference)		1(Reference)	
Positive	3.788(1.377–10.421)		3.133(1.537–6.384)		5.487(1.844–16.326)		3.082(1.530–6.206)	
Postoperative complications		0.238				0.162		
Yes	1(Reference)				1(Reference)			
No	2.247(0.586–8.611)				2.677(0.673–10.651)			
Postoperative chemotherapy		0.004		0.0006		0.0001		0.0001
Yes	1(Reference)		1(Reference)		1(Reference)		1(Reference)	
No	4.021(1.552–10.422)		3.056(1.612–5.792)		7.301(2.626–20.301)		3.876(1.944–7.730)	
Postoperative radiotherapy		0.750				0.382		
Yes	1(Reference)				1(Reference)			
No	1.181(0.424–3.294)				1.638(0.542–4.948)			

*FAR, fibrinogen-to-albumin ratio; DFS, disease-free survival; OS, overall survival: CI, confidence interval; BMI, body mass index; ALB, albumin; FIB, fibrinogen; ALT, alanine transaminase; AST, aspartate transaminase; ALP, alkaline phosphatase; CEA, carcinoembryonic antigen; CA, cancer antigen; FDP, fibrin degradation product; CK, creatinine kinase; EGFR, epidermal growth factor receptor.*

### Survival and Prognosis

**Figure 2:**
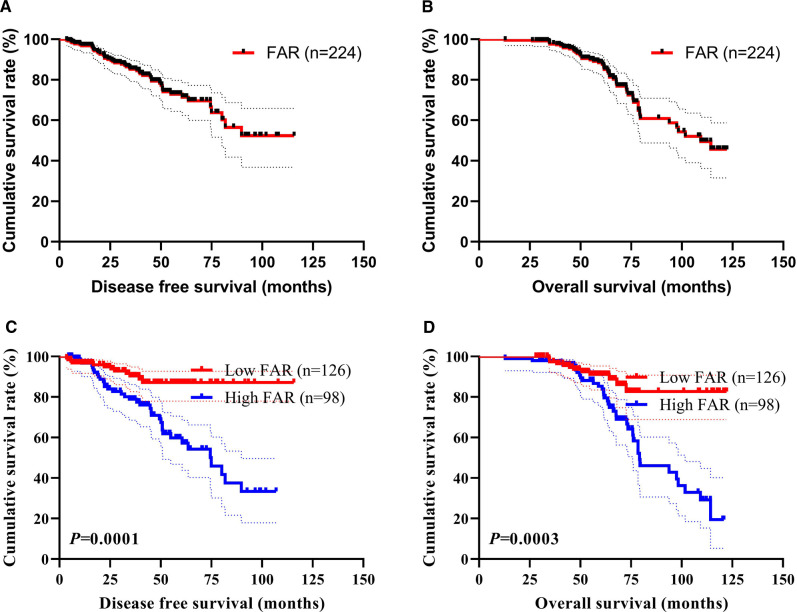
Disease free survival (DFS) and overall survival (OS) in triple-negative breast cancer (TNBC). (**A**) All enrolled patients for DFS, (**B**) All enrolled patients for OS, (**C**) Correlation of FAR with DFS, (**D**) Correlation of FAR with OS. Statistical analyses were performed using log-rank tests.

The median DFS and OS was 39.90 and 60.37 months, respectively. FAR shown significant prognostic association with DFS (*P* = 0.0001, HR: 3.395, 95% CI, 1.763–6.538; *P* < 0.0001, HR: 4.600, 95% CI, 2.201–9.616) and OS (*P* = 0.0006, HR: 3.166, 95% CI, 1.641–6.111; *P* < 0.0001, HR: 5.555, 95% CI, 2.472–12.481). The median DFS and OS were 42.58 vs. 63.64 months, and 38.02 vs. 57.72 months, for the low and high FAR groups, respectively. Compared with the high FAR group, the median DFS and OS in the low FAR group were significantly longer (*χ*^2 ^= 15.080, *P* = 0.0001; *χ*^2 ^= 13.140, *P* = 0.0003). The results were shown in Figure 2.

### Establishment of the Nomogram

**Figure 3:**
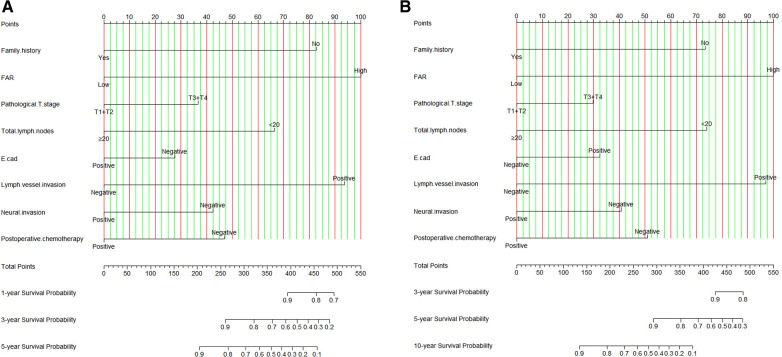
FAR based nomogram for predicting disease free survival (DFS) and overall survival (OS). (**A**) FAR based nomogram for predicting DFS; (**B**) FAR based nomogram for predicting OS.

**Figure 4:**
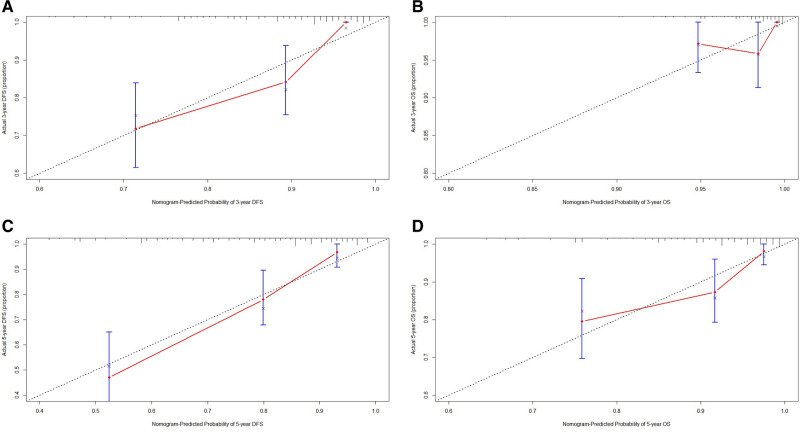
The calibration curves for predicting the 3-, 5-year DFS and OS rates. (**A**) The calibration curves for predicting the 3-year DFS rate in patients with triple-negative breast cancer (TNBC); (**B**) The calibration curves for predicting the 3-year OS rate in patients with TNBC; (**C**) The calibration curves for predicting the 5-year DFS rate in patients with TNBC; (**D**) The calibration curves for predicting the 5-year OS rate in patients with TNBC.

Though the results of the multivariate Cox proportional hazards model, we constructed an novel nomogram for DFS and OS. In this nomogram, each variable was imputed a weighted point, and the sum of the points can predict 1-, 3-, and 5-year survival probabilities for DFS, and 3-, 5-, and 10-year survival probabilities for OS. A higher patient grade was associated with a lower survival probability. The nomogram for DFS and OS had unique features, and integrated family history, FAR, pathological T stage, total lymph nodes, E-cad status, LVI, neural invasion, and postoperative chemotherapy (Figure 3). Moreover, we also used the calibration curve to evaluate the nomogram for predicted and the actual probability of DFS and OS. The prediction line matched the reference line well for postoperative 3- and 5-year DFS and OS, showing good performance of the nomogram (Figure 4).

### Correlation Between FAR and Menopause

Overall, 93 and 131 patients were post-menopausal and pre-menopausal, respectively. To investigate the prognostic efficiency of the FAR, a reanalysis according to menopause status was conducted. Pre-menopausal patients survived longer than post-menopausal patients (*χ*^2 ^= 7.372, *P* = 0.007; *χ*^2 ^= 5.115, *P* = 0.024). The low FAR post-menopausal patients survived significantly longer than the high FAR group (*χ*^2 ^= 7.123, *P* = 0.008; *χ*^2 ^= 6.512, *P* = 0.011). The low FAR pre-menopausal patients survived significantly longer than their high FAR counterparts (*χ*^2 ^= 5.023, *P* = 0.025; *χ*^2 ^= 4.879, *P* = 0.027). The results were shown in Supplementary Figure S2.

### The Prognostic Significance of FAR in TNBC Patients by Pathological TNM Stage

Further, we determined the correlation of FAR with pathological TNM stage, including 119 (53.13%) and 105 (46.87%) patients diagnosed with pathological stages I + II and III, respectively. Not surprisingly, patients with pathological stages I + II survived longer compared to those with pathological stage III (*χ*^2 ^= 4.840, *P* = 0.028; *χ*^2 ^= 7.558, *P* = 0.006). Among patients with pathological stages I + II, the low FAR group survived significantly longer than the high FAR group (*χ*^2 ^= 10.280, *P* = 0.001; *χ*^2 ^= 8.075, *P* = 0.005). Among patients with pathological stage III, the low FAR group survived significantly longer than the high FAR group (*χ*^2 ^= 3.619, *P* = 0.057; *χ*^2 ^= 3.438, *P* = 0.064). The results were shown in Supplementary Figure S3.

### Correlation Between FAR and Lymph Vessel Invasion

From the univariate and multivariate analyses, LVI was a prognostic factor of DFS (*P* < 0.0001, HR: 4.992, 95% CI, 2.295–10.858; *P* < 0.0001, HR: 3.865, 95% CI, 2.237–6.681) and OS (*P* < 0.0001, HR: 5.582, 95% CI, 2.374–13.125; *P* < 0.0001, HR: 4.893, 95% CI, 2.675–8.950). To investigate the prognostic efficiency of FAR, reanalysis according to LVI was performed. Overall, 76 (33.93%) and 148 (66.07%) patients were diagnosed with and without LVI, respectively. Patients without LVI survived longer than those with LVI (*χ*^2 ^= 8.851, *P* = 0.003; *χ*^2 ^= 14.620, *P* = 0.0001, respectively). Among patients without LVI, the low FAR group survived significantly longer than their high FAR counterparts (*χ*^2 ^= 18.600, *P* < 0.0001; *χ*^2 ^= 15.990, *P* < 0.0001). Among patients with LVI, the low FAR group survived significantly longer than their high FAR counterparts (*χ*^2 ^= 0.011, *P* = 0.918; *χ*^2 ^= 0.222, *P* = 0.638). The results were shown in Supplementary Figure S4.

### Chemotherapy and Radiotherapy after Operation

From the univariate and multivariate analyses, chemotherapy was a prognostic factor of DFS (*P* = 0.004, HR: 4.021, 95% CI, 1.552–10.422; *P* = 0.001, HR: 3.056, 95% CI, 1.612–5.792) and OS (*P* = 0.0001, HR: 7.301, 95% CI, 2.626–20.301; *P* = 0.0001, HR: 3.876, 95% CI, 1.944–7.730). Overall, 147 (65.63%) and 77 (34.37%) patients received or did not receive chemotherapy, respectively. Patients who received chemotherapy after surgery survived longer than those that did not receive chemotherapy (*χ*^2 ^= 5.027, *P* = 0.025; *χ*^2 ^= 4.510, *P* = 0.034). Among patients who did not receive chemotherapy, the low FAR group survived longer than the high FAR counterparts (*χ*^2 ^= 5.858, *P* = 0.016; *χ*^2 ^= 3.762, *P* = 0.052). In patients who received chemotherapy, survival was significantly longer in the low FAR group than in their high FAR counterparts (*χ*^2 ^= 9.367, *P* = 0.002; *χ*^2 ^= 9.203, *P* = 0.002).

Moreover, we also analyzed the correlation between FAR and radiotherapy; and 181 (80.80%) and 43 (19.20%) patients received or did not receive radiotherapy after operation, respectively. Patients who received radiotherapy after surgery survived longer than those who did not (*χ*^2 ^= 1.727, *P* = 0.189; *χ*^2 ^= 4.542, *P* = 0.033, respectively). Among patients who did not receive radiotherapy, those in the low FAR group survived longer than their high FAR group counterparts (*χ*^2 ^= 4.465, *P* = 0.035; *χ*^2 ^= 2.613, *P* = 0.106). Among patients who received radiotherapy, those in the low FAR group survived longer than their high FAR group counterparts (*χ*^2 ^= 9.935, *P* = 0.002; *χ*^2 ^= 8.837, *P* = 0.003).

## Discussion

TNBC is characterized by high histological grade and invasiveness; however, there is still a lack of effective treatment (21). In breast cancer development, inflammation plays an important role by promoting tumor occurrence and progression, and can also affect proliferation, invasion, apoptosis, and angiogenesis of tumor cells, and inhibit anti-tumor immune response (22, 23). Some studies have indicated FIB as a multifunctional protein, usually accompanied by tissue abnormalities, infection, and inflammation (24). Moreover, coagulation system activation and coagulation factor release play important roles in the process of tumor occurrence and progression (25). Hyperfibrinogenemia increases tumor progression and inhibits tumor cell immunity by natural killer cell-mediated cytotoxicity (26). In addition to reflecting the nutritional status of the host, serum ALB can also be affected by inflammatory factors, which in turn reflects the level of inflammation in the body. ALB, which is used for tissue repair and as a carrier protein mainly, is also used assessing metabolism and immunity. Furthermore, in patients with hypoproteinemia, the occurrence of tumor cachexia would be aggravated, and the nutritional status will further deteriorate (27). The inflammatory immune parameters, such as FIB, systemic inflammation response index (SIRI), systemic immune-inflammation index (SII), and C-reactive protein (CRP), were included to assess breast cancer prognosis, and parameters with high values were independent prognostic risk factors (28–31). Recently, FAR, proven to be related to prognosis in various malignant tumors, has become a key prognostic factor (32, 33). Therefore, it is important to further assess the clinical prognosis in TNBC.

In this study, 224 TNBC patients were enrolled and retrospectively analyzed. The results indicated FAR as a prognostic factor, and the median DFS and OS in the low FAR group were longer than those in the high FAR group. Zheng et al. shown that combined preoperative fibrinogen-albumin ratio and platelet-lymphocyte ratio score (FAR-PLR score) was a potential new biomarker for predicting survival and prognosis of breast cancer, and may facilitate better clinical decision making for breast cancer treatment by the physicians (34). Cao et al. indicated that patients with preoperative high FAR were prone to short progression-free survival and low OS rate, and that preoperative FAR was a potential prognostic factor of breast cancer (35). Another study shown that preoperative plasma FIB was independently associated with poor prognosis and might serve as a valuable parameter for risk assessment in patients with breast cancer, especially in stage II-III, Luminal subtypes and TNBC patients (36). Moreover, we constructed a nomogram based on the potential prognostic factors by the multivariate analysis. The nomogram predicts the 1-, 3-, and 5-year survival probabilities of DFS, and the 3-, 5-, and 10-year survival probabilities of OS. Moreover, the prediction line matches the reference line well for postoperative survival DFS and OS survival by calibration curves.

Early menarche and late menopause increase the risk of breast cancer in women. Our study indicated that patients with pre-menopausal status survived longer than those with post-menopausal status, and patients with low FAR survived longer than those with high FAR. The underlying mechanism could be related to the use of hormone therapy in postmenopausal women with a greater increased risk of ER-positive than ER-negative tumors; moreover, young age at menarche and older age at menopause increase breast cancer risk (37). Further, we determined the correlation of FAR with pathological TNM stage, and shown that patients with pathological stages I + II survived longer than the pathological stage III patients, while patients with low FAR survived longer than the high FAR group. Commonly, the lymphatic vessel density and lymphovascular invasion are evaluated to identify the clinicopathological outcomes in breast cancer (38). We also analyzed the correlation between FAR and LVI and found LVI to be a prognostic factor, and patients without LVI survived longer than those with LVI. Kurozumi et al. found that LVI was associated with metastasis development in invasive breast cancer as well as with a specific transcriptomic profile of potential prognostic value (39). Another study reported a strong association of LVI with both breast cancer-specific survival (BCSS) and distant metastasis-free survival (DMFS). LVI, a strong predictor of patients’ outcome in invasive breast cancer, should be included in the breast cancer staging systems (40).

Some of the potential mechanisms to explain the clinical significance of FAR in breast cancer include the following. FIB is upregulated by the inflammatory cytokine, IL-6, and has been demonstrated to mediate cell proliferation and form an extracellular matrix that binds to tumor cell surfaces, further increasing cancer cell adhesion, invasion, and metastasis (41–43). Serum ALB, a common indicator of nutritional status, is related to the immune status and prognosis of breast cancer. Moreover, tumor necrosis factor (TNF)-*α* selectively inhibits ALB gene expression and reduces ALB level ultimately (44, 45). FAR is a more comprehensive serum marker, reflecting the inflammation and nutritional status of patients with tumors, and acting as a new promising prognostic indicator (46). The FAR and fibrinogen-prealbumin ratio (FPR) for patients with cancer were assessed in a meta-analysis. They reported correlation of a low FAR and high FPR with increased risk of cancer mortality and recurrence and might be promising prognostic markers for malignant tumors (25).

There were several limitations to this study. First, this was a retrospective study, and a small number of patients were included; therefore, more patients should be enrolled for further study. Second, peripheral blood parameters were not compared with inflammatory parameters for the primary tumor tissue. Third, power calculation for an estimation of the sample size used for the study was not done. Finally, some heterogeneities were observed in the patients’ treatment approaches after surgery, which might have led to a different clinical prognosis. Thus, large-scale, multicenter, prospective studies should be conducted to further assess the prognostic role of FAR and determine the high-risk population of patients with breast cancer.

## Conclusion

In conclusion, FAR is significantly associated with the survival and prognosis of TNBC. It is a strong and independent factor and is of great significance for identifying high-risk patients and providing accurate treatment. Furthermore, FAR is a simple, objective, and inexpensive biomarker for preoperative clinical evaluation, and is potentially applicable in clinical practice.

## Data Availability

The raw data supporting the conclusions of this article will be made available by the authors, without undue reservation.
