# Supplementary figures and images for: The Prognostic Significance of the Fibrinogen-to-Albumin Ratio in Patients With Triple-Negative Breast Cancer: A Retrospective Study
Source: Front Surg. 2022 Jun 14;9:916298. doi: 10.3389/fsurg.2022.916298 (PMC9237393; doi:10.3389/fsurg.2022.916298)

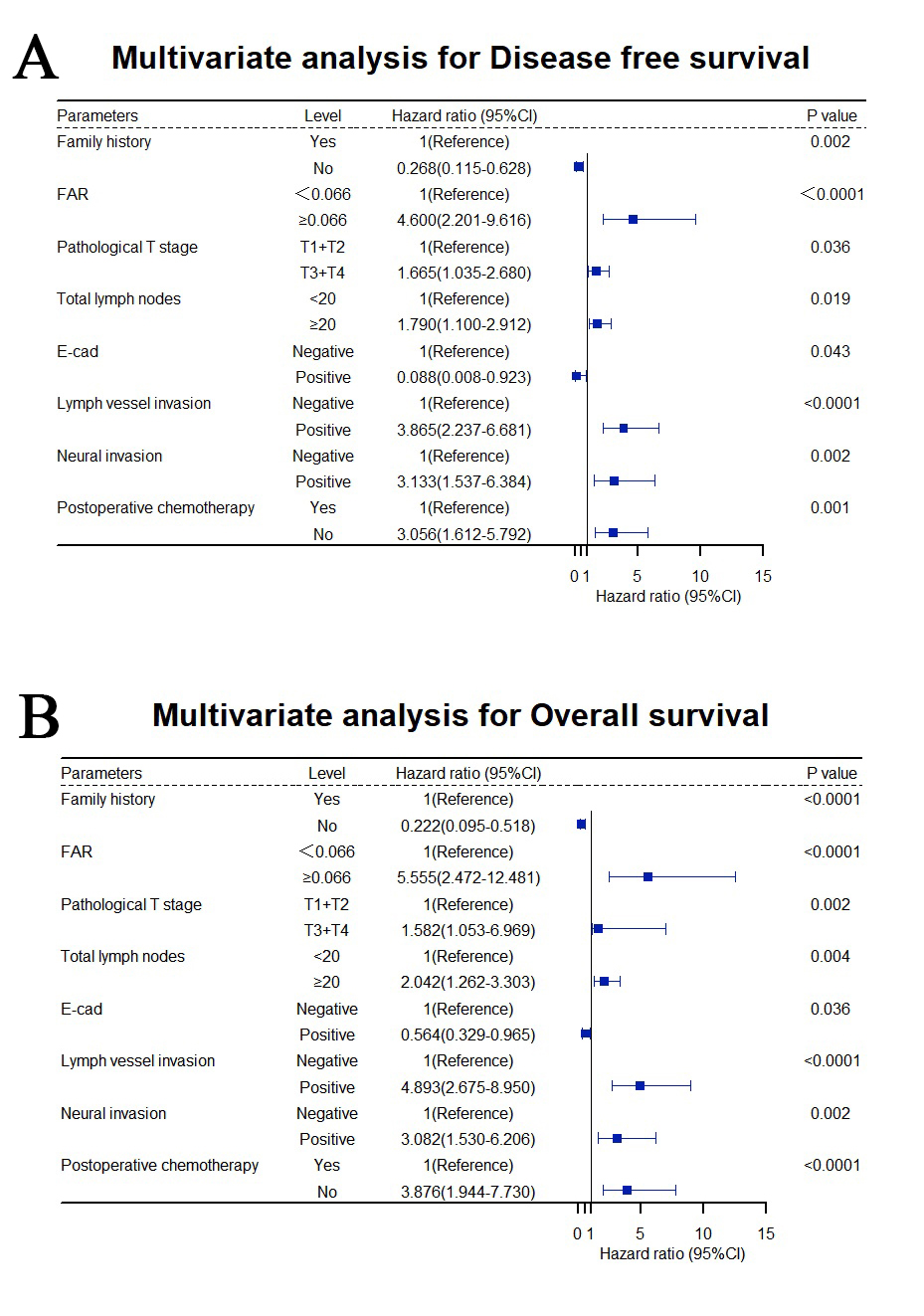

Supplement: Supplementary file 1 [file Figure_5_v1.jpeg]

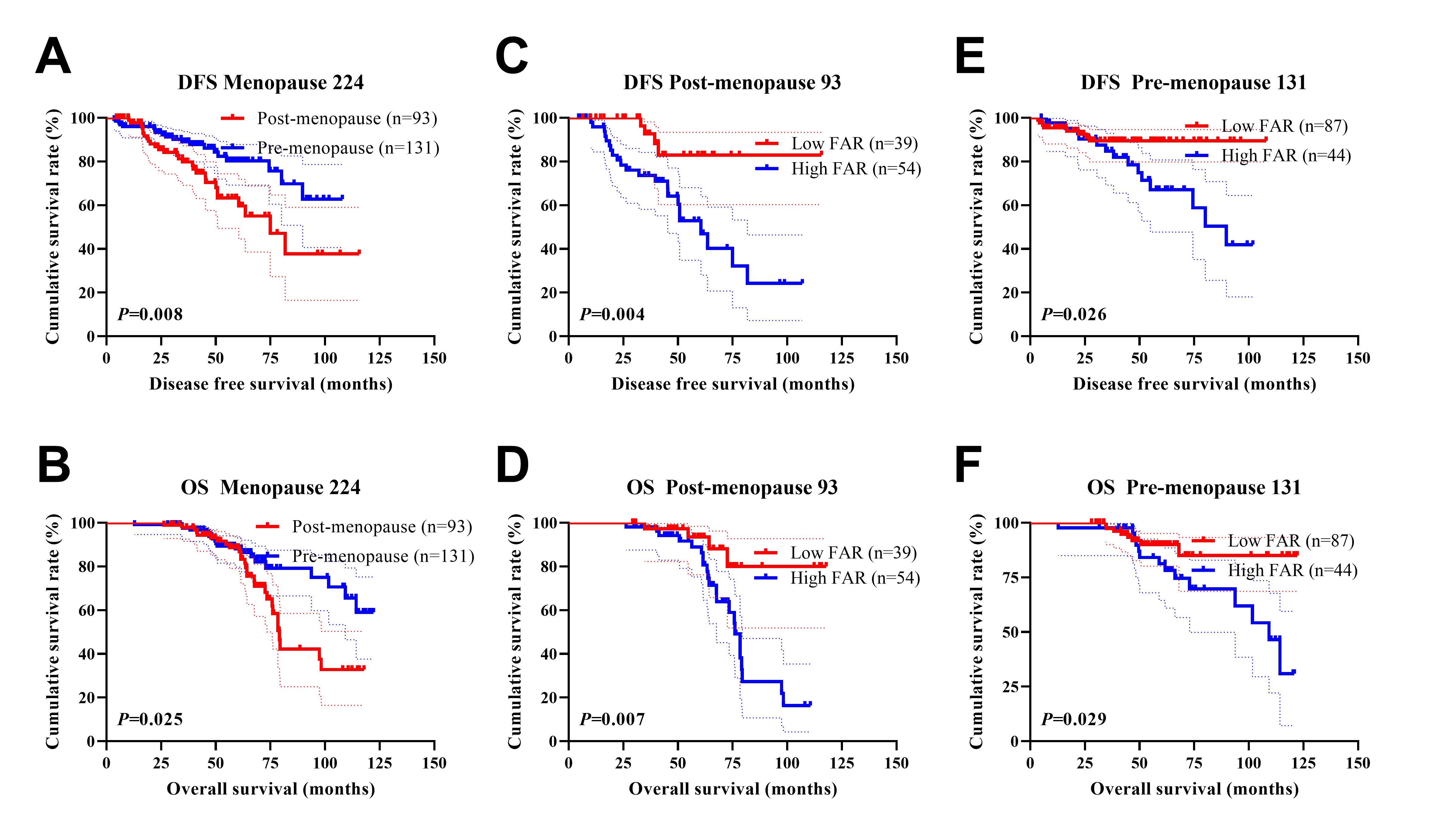

Supplement: Supplementary file 2 [file Figure_6_v1.jpeg]

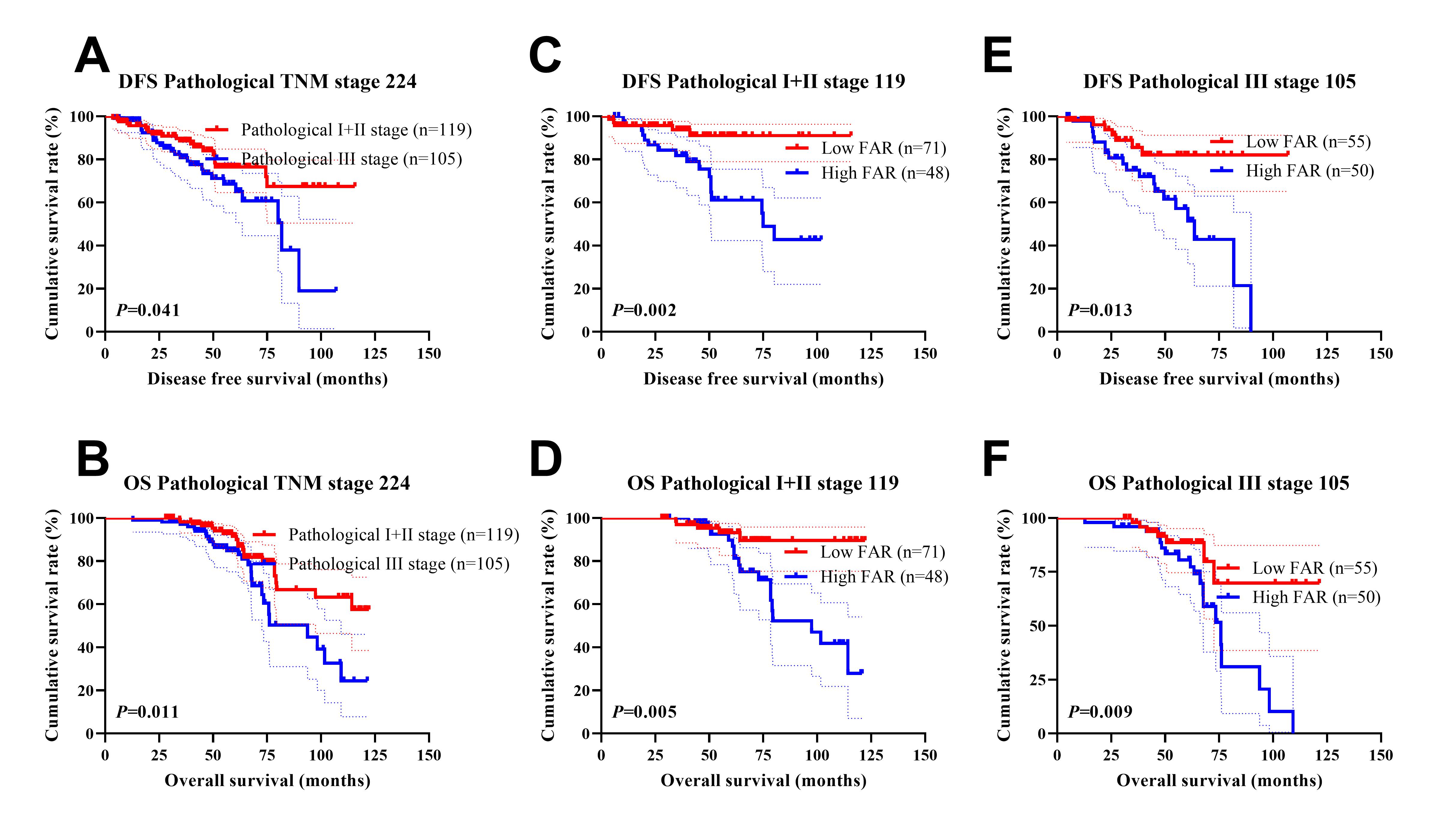

Supplement: Supplementary file 3 [file Figure_7_v1.jpeg]

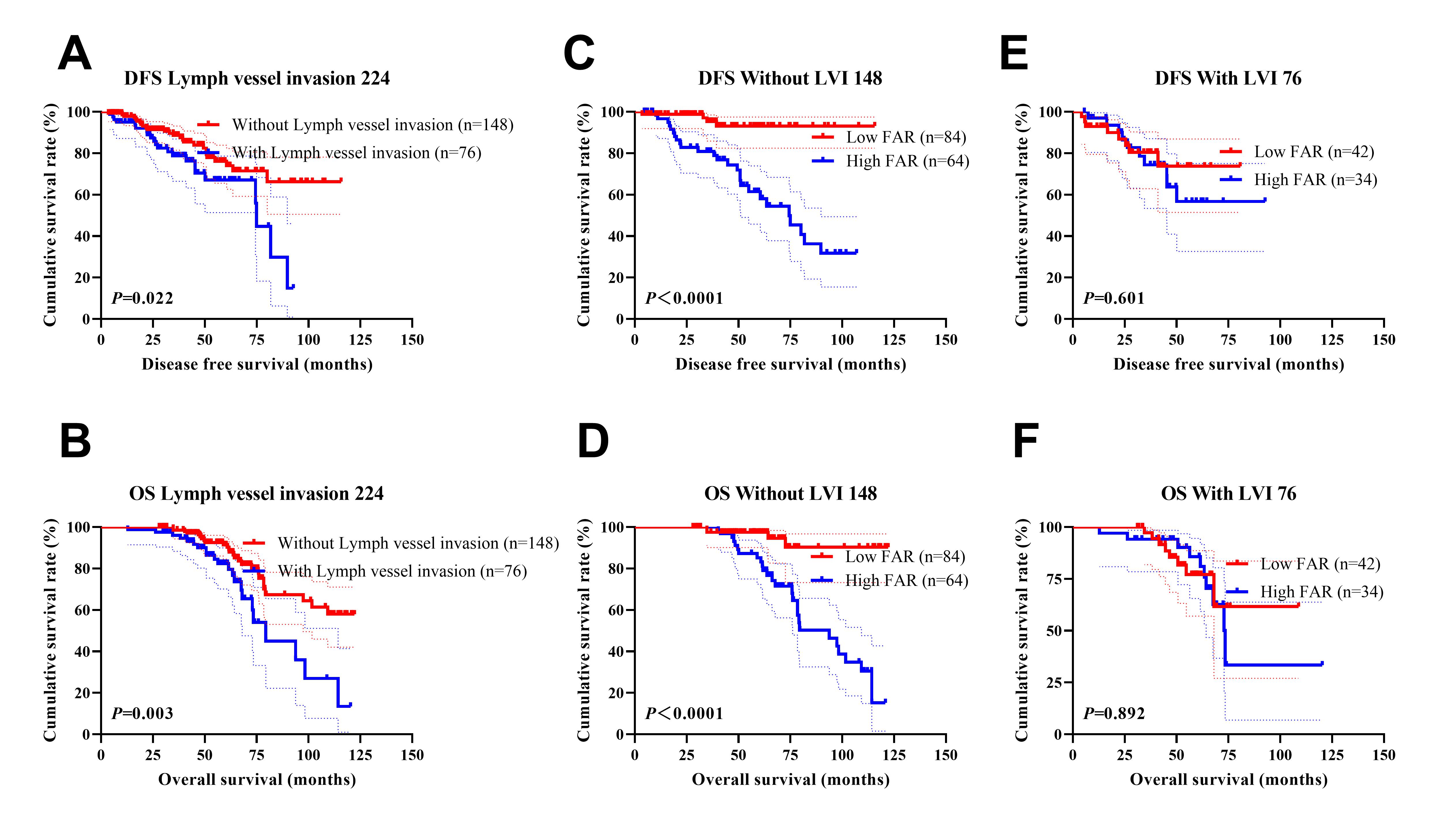

Supplement: Supplementary file 4 [file Figure_8_v1.jpeg]
